# Refill Adherence in Relation to Substitution and the Use of Multiple Medications: A Nationwide Population Based Study on New ACE-Inhibitor Users

**DOI:** 10.1371/journal.pone.0155465

**Published:** 2016-05-18

**Authors:** Pernilla J. Bjerkeli, Anna K. Jönsson, Eva Lesén, Ann-Charlotte Mårdby, Karolina Andersson Sundell

**Affiliations:** 1 Nordic School of Public Health, Gothenburg, Sweden; 2 Unit for Social Epidemiology, Clinical Research Centre, Faculty of Medicine, Lund University, Lund, Sweden; 3 Department of Forensic Genetics and Forensic Toxicology, National Board Forensic Medicine, Linköping, Sweden; 4 Nordic Health Economics AB, Gothenburg, Sweden; 5 Research and Development, Sahlgrenska University Hospital, Gothenburg, Sweden; 6 Section for Epidemiology and Social Medicine, Department of Public Health and Community Medicine at Institute of Medicine, Sahlgrenska Academy at University of Gothenburg, Gothenburg, Sweden; Taipei Veterans General Hospital, TAIWAN

## Abstract

**Objective:**

Generic substitution has contributed to economic savings but switching products may affect patient adherence, particularly among those using multiple medications. The aim was to analyse if use of multiple medications influenced the association between switching products and refill adherence to angiotensin-converting-enzyme (ACE) inhibitors in Sweden.

**Study Design and Setting:**

New users of ACE-inhibitors, starting between 1 July 2006 and 30 June 2007, were identified in the Swedish Prescribed Drug Register. Refill adherence was assessed using the continuous measure of medication acquisition (CMA) and analysed with linear regression and analysis of covariance.

**Results:**

The study population included 42735 individuals whereof 51.2% were exposed to switching ACE-inhibitor and 39.6% used multiple medications. Refill adherence was higher among those exposed to switching products than those not, but did not vary depending on the use of multiple medications or among those not. Refill adherence varied with age, educational level, household income, country of birth, previous hospitalisation and previous cardiovascular diagnosis.

**Conclusion:**

The results indicate a positive association between refill adherence and switching products, mainly due to generic substitution, among new users of ACE-inhibitors in Sweden. This association was independent of use of multiple medications.

## Introduction

The World Health Organisation defines adherence as the extent to which a person’s behaviour—taking medication, following a diet, and/or executing lifestyle changes—corresponds with agreed recommendations from a health care provider [1]. Only around half of persons with chronic disorders are adherent to prescribed medication regimens [2, 3]. Low adherence is associated with increased morbidity and mortality and is costly to manage [1, 4]. Adherence can be measured in several ways, one is to use register data of filled prescriptions to assess refill adherence [5]. Refill adherence is a useful measurement, in particular in settings where universal drug coverage applies, such as Sweden [5].

Another factor that can affect how prescribed medications are used is generic substitution. Generic substitution takes place when a different product containing the same active substance substitutes a prescribed drug. Sweden introduced mandatory generic substitution in 2002 and today, approximately 40% of all dispensed prescriptions are subject to generic substitution [6]. In Sweden, and many other countries, mandatory generic substitution means that at each prescription fill, the pharmacist dispenses the cheapest available equivalent alternative to the prescribed drug to the patient. A consequence of this is that the patient may receive a different product from one purchase to the other. If the patient wishes to purchase the same product at each fill he or she has to pay the price difference between the cheapest available product and that requested out of pocket.

Although generic substitution has contributed to substantial economic savings for the health care system [7], there are also reports of negative outcomes for the patients [8–10]. Interview based studies indicate that patients find it demanding to manage their medications after generic substitution [11–13]. They do, for example, find it difficult to keep track of which medications contain the same active ingredient. Despite these findings, previous studies report conflicting results whether generic substitution affects medication adherence negatively or not. Some studies reported a negative association between adherence and generic substitution [9], whereas others reported no such effects [14]. Other studies reported a higher adherence among those exposed to generic substitution [15–17].

Several studies investigating the potential negative outcomes of generic substitution suggest that the problems are greatest for patients using many different medications [8, 10, 11, 18]. A patient group often using multiple medicines is patients with hypertension. Angiotensin-converting-enzyme (ACE)-inhibitors are one of the most commonly used types of antihypertensives [19], and they are included in treatment guidelines for hypertension in Europe as well as the United States [20, 21]. Also, there are several generic alternatives available. Users of ACE-inhibitors are thus a suitable group to study use of multiple medications and substitution.

Benner et al showed that adherence among hypertensive patients is negatively associated with the number of prescribed antihypertensive medications [3], thus highlighting the importance of considering the use of multiple medications when analysing adherence. Van Wijk et al found no negative effect on adherence following generic substitution of antihypertensive drugs [16]. Considering the previously mentioned reports suggesting more pronounced difficulties associated with generic substitution among patients using multiple medications [8, 10, 11, 18], it is important to focus on adherence in this group. We thus analysed if use of multiple medications influenced the association between switching products, as a consequence of substitution and therapeutic switching, and refill adherence to ACE-inhibitors.

## Methods

### Study population and study period

The study population included all new users of ACE-inhibitors in Sweden with a first purchase (date of the first purchase is the index date) of an ACE-inhibitor (Anatomical Therapeutic Chemical Classification System (ATC)-code C09A) between 1 July 2006 and 30 June 2007, who had not filled any prescriptions of an ACE-inhibitor during the preceding 12 months. Individuals were followed until emigration, death or for a maximum of two years after their index date, whichever occurred first.

Since switching between different ACE-inhibitors is part of clinical practice we did not exclude those who switched between different ACE-inhibitors during the study period. Refill adherence was only calculated for ACE-inhibitors, therefore those switching from ACE-inhibitors to an angiotensin receptor blocker (ARB, ATC-code C09B) were censored on the day of the first ARB purchase. Individuals aged less than 18 years at the index date, individuals receiving multi-dose dispensed drugs during the study period, or those with non-interpretable dosage texts in filled prescriptions of ACE-inhibitors were excluded.

### Data

Data on purchased prescription medicines were collected from the Swedish Prescribed Drug Register (SPDR) [22]. The register encompasses information about the dispensed medicine (product, ATC-code, amount, cost, date of issue and purchase, and dosage as free text), the prescriber (type of care unit, ownership), and the patient (age, sex, place of residence). Information on hospital care episodes (hospitalisation and previous cardiovascular diagnosis) and socioeconomic characteristics (educational level, household income and country of birth) in the calendar year before the index date were collected by record linkage to the National Patient Register and the Longitudinal integration database for health insurance and labour market studies (LISA), respectively, using the unique personal identification number that all Swedish residents hold.

Ethical approval is a prerequisite for obtaining register data. Such an approval was received for the Refill Adherence in Registers (RARE) project [23–25], which this study is part of, from the Regional Ethical Review Board in Gothenburg, Sweden (No 284–09). In concordance with this approval, no informed consent was obtained since the study only includes national register data. The RARE-project is a research project, which uses a large database containing data from Swedish registers to analyse different aspects of refill adherence [23–26].

### Exposure

Exposure to switching products was defined as receiving a different ACE-inhibitor product (with the same active ingredient and strength) than the one that was dispensed at the previous purchase. The reason for such a switch would most likely be generic substitution in the majority of the cases but it could also be the result of the prescriber for some reason issuing a new prescription with a different product. According to this definition, it did not matter whether the patient received a brand or a generic product. It only mattered whether the product was different from what the patient received at his or her previous purchase. This definition was chosen since the switching between different products is most likely the aspect of generic substitution that is most evident to the patient. At the time of the data collection, there were generic alternatives available for all ACE-inhibitors on the Swedish market. Receiving a parallel imported medicine with the same ingredient was not considered a substitution nor was receiving a different strength of the same brand.

Use of multiple medications was defined as the total number of different other medicines purchased by the individual in the year before the index date. These were grouped into up to five other medicines, and more than five other medicines.

### Outcome measure

Adherence to ACE-inhibitors was assessed using the continuous measure of medication acquisition (CMA) [27, 28]. CMA measures the proportion of days’ in the study period that are covered by the supply of medication purchased by the patient during the same period and is calculated as follows [23]:

$$\mathrm{CMA}=\frac{\mathrm{Number of days supply of ACE inhibitor dispensed during the study period}}{\mathrm{Number of days in the study period}}$$

A CMA approaching 100% indicates that that patient has purchased the exact same amount of medication as he or she is supposed to use during the study period, according to the dosage text on the prescriptions. Values below 100% indicate that the patient has purchased less medication than recommended and values exceeding 100% indicate that the patient has bought more than recommended. CMA was therefore set to a maximum of 100% to reflect whether the patient had medications at hand during the whole study period.

In the present study, CMA was assessed for each individual starting from index date until end of follow up. Number of days’ supply was based on the amount purchased at each fill divided by the daily dose. Daily dose was determined by coding the dosage texts available in free text format using an algorithm developed by the research group [23]. For this study, the algorithm was modified by the first author and one of the co-authors (AKJ). To assess the accuracy of the interpretations, a random selection of 10% of the 26000 unique dosage text strings were checked by one of the co-authors. To be considered acceptable a concordance of at least 95% was required a priori. The result of the validation was a concordance of 96%.

Titration was taken into account when interpreting dosage texts. The dosage during the titration period was calculated as half the maintenance dose. If the exact same titration occurred more than once for the same patient during the study period, only the first time was taken into account. Prescriptions are commonly iterated and the same dosage text may be recorded at every purchase even though it is clear to the patient that the titration should only be done the first time.

There is no information on medication use during hospitalisation in the SPDR. By linking the SPDR to the National Patient Register we could identify periods of hospitalisation and omit these from the study period.

### Covariates

Number of generic substitutions during the year before index date was assessed for all medicines purchased during this period. Here a substitution was defined based on the substitution variable in the SPDR that denotes whether a prescribed product was substituted to a generic product.

Purchased anti-diabetic medications in the year before the index date was used as an indicator of pharmacologically treated diabetes and divided into four groups; none, oral anti-diabetics only (ATC-code A10B), oral anti-diabetics and insulin (ATC-codes A10A and A10B), and insulin only (ATC-code A10A). Purchased cardiovascular (CVD) medication in the same period was used as an indicator of previous pharmacological treatment for CVD (ATC-groups B01 and C01-C10).

Previous CVD, an indicator for secondary prevention, was defined as having a diagnosis for cardiovascular disease registered in the National Patient Register within five years before the index date. Cardiovascular diseases included ischaemic heart disease (ICD-10 code I20-I25), pulmonary heart disease and diseases of pulmonary circulation (I26-I28), cerebrovascular diseases (I60-I69), diseases of arteries, arterioles and capillaries (I70-I79). Hospitalisation for any cause during the year prior to the index date was categorized into a dichotomous variable.

Educational level was grouped into four categories: elementary school, high school, higher education for two years or less and higher education for more than two years. Household income was adjusted for number of individuals in the family (household income divided by the square root of number of individuals in the family) and then divided into quartiles. Country of birth was categorised as follows: Sweden, other Nordic countries, the rest of Europe and the rest of the world.

### Sensitivity analyses

The following sensitivity analyses were performed in order to validate the definitions used in this study. First, as the definition of switching products relies on which product the individual received at his/her previous purchase; the study population was restricted to include only those who purchased an ACE-inhibitor at least twice during the study period. In the second sensitivity analysis, we restricted the study population to include only those with no switches between ACE-inhibitors containing different active ingredients during the study period.

The third sensitivity analysis tested the effect of an altered definition of substitution and switching products of the ACE-inhibitor. In the main analysis it was defined as a purchase where the individual received another product than at his/her previous purchase. In this sensitivity analysis it was defined as a purchase where the individual received another product than what was written on the prescription the covariate describing the number of generic substitutions.

In a fourth sensitivity analysis, we investigated how time from index date to the first occurrence of switching products affected the outcome variable. We presented the mean CMA and standard deviation by time from index date to the first time of switching products and use of multiple medicines, respectively. Time from index date was categorized as: up to 4 months (0–120 days), 4–8 months (121–240 days), 8–12 months (241–365 days) and at least 12 months (366 and above days). The time frame four months was chosen since most patients obtain packages with 100 tablets and considering that many patients titrate the doses during the first months, the first supply is likely to last 3.5–4 months. To further investigate this, time to the first switching of products was included as a continuous variable in the multiple linear regression model.

### Statistical analyses

Two sample t-test was used to compare CMA across dichotomous variables such as exposure to switching products, use of multiple medications, sex, use of CVD medication, hospitalization and previous cardiovascular diagnosis. Analysis of variance (ANOVA) was used to compare CMA across class variables such as age, educational level, country of birth, household income, use of diabetes medication and number of substitutions of other medications. When a statistically significant difference was found the Tukey post hoc test was used to locate the difference. The significance level was set to p<0.05. Analysis of co-variance (ANCOVA) was used to analyse if CMA differed by switching products and use of multiple medicines when these variables were combined into four mutually exclusive groups, controlling for potential confounders in the same way as in the multiple linear regression described below.

Multiple linear regression was used to analyse the relationship between CMA, switching products, use of multiple medications, sex, age, educational level, household income, country of birth, use of other CVD medication, use of diabetes medication, number of substitutions on other medications, hospitalisation and previous cardiovascular diagnosis. First single linear regressions were made for all independent variables, with CMA as dependent variable. Age, sex and independent variables with a p-value < 0.1 were included in the final multiple model. Residual plots were used to assess normality. All included variables were checked for colinearity, a maximum variance inflation factor of 10 was considered acceptable.

All statistical analysis was conducted using SAS statistical software version 9.3 (SAS Institute, Cary, NC, USA) and Stata/SE version 13 (Statacorp, Texas, USA).

## Results

### Study population

The final study population encompassed 42735 individuals (Table 1).

**Table 1:** Exclusion of identified new users of ACE-inhibitors according to predefined exclusion criteria.

Reason for exclusion	Number excluded	Number of new users of ACE-inhibitors
Starting number		48054
Purchase of multi dose dispensed drug	2461	45593
Death or migration before index date	109	45484
Non-interpretable dosage text	2726	42758
Zero days in the observation period*	23	**42735**

* After adjusting the length of the study period for individuals who died, emigrated and/or switched to an Angiotensin II receptor antagonist during the study period.

Table 2 shows background characteristics of the study population. Mean age was 65.3 years (S.D. 13.1) and 49.5% were women. Fifteen percent were born outside of Sweden. Around half of the study population had been exposed to switching of the ACE-inhibitor during the study period (Table 2). In the year before the index date, 39.6% had purchased more than five different prescription medications and 14.2% had experienced substitution of these medications at least five times. Over one-quarter had been hospitalized at some time during the year prior to the index date and almost one fifth had previous CVD.

**Table 2:** Continuous measure of Medication Acquisition (CMA) for different groups of new users of ACE-inhibitors (n = 42735).

Variable	n	(%)	CMA (%)	p-value
**Total**	**42735**	**100**	**53.5**	**-**
**Switching products**				
Yes	21872	51.2	81.0	<0.0001
No	20863	48.8	24.6	
Number of other medicines*				
0–5	25823	60.4	55.6	<0.0001
>5	16912	39.6	50.3	
**Sex**				
Men	21559	50.5	57.8	<0.0001
Women	21176	49.5	49.1	
**Age (years)**				
< 45	2403	5.62	49.0^a^	<0.0001
45–54	5434	12.7	51.7^a^^,^^b^	
55–64	10879	25.5	53.0^a^^,^^c^	
65–74	11519	27.0	53.4^a^^,^^d^	
≥75	12500	29.3	55.7^a^^,^^b^^,^^c^^,^^d^	
**Educational level**				
Elementary school	10666	33.3	53.6^a^	0.0115
High school	14568	45.5	52.7	
Higher education ≤2 years	1001	3.1	53.6	
Higher education >2 years	5756	18.0	51.5^a^	
Missing	10744	-	-	
**Household income**				
1^st^ quartile (lowest)	10632	25.0	52.5^a^	<0.0001
2^nd^ quartile	10633	25.0	52.7^b^	
3^rd^ quartile	10642	25.0	53.6	
4^th^ quartile	10634	25.0	54.9^a^^,^^b^	
Missing	194	-	-	
**Country of birth**				
Sweden	36020	84.7	54.0^a^^,^^b^^,^^c^	<0.0001
Nordic countries	2471	5.8	51.5^a^	
Europe	2450	5.8	50.0^b^	
Rest of the world	1600	3.8	50.5^c^	
Missing	194	-	-	
**Previous cardiovascular diagnosis**				
Yes (within 5 years)	7725	18.1	61.1	<0.0001
No	35010	81.9	51.8	
**Hospitalization**				
Yes (during the previous year)	11885	27.8	59.1	<0.0001
No	30850	72.2	51.3	
Use of other cardiovascular medicine*				
Yes	25949	60.7	50.8	<0.0001
No	16786	39.3	57.5	
Use of diabetes medicines*				
None	37078	86.8	52.8^a^^,^^b^^,^^c^	<0.0001
Oral antidiabetics only	2922	6.8	58.1^a^	
Oral antidiabetics and Insulin	1183	2.8	60.9^b^^,^^d^	
Insulin only	1552	3.6	56.0^c^^,^^d^	
Number of substitutions on other medicines*				
0	18496	43.3	48.5^a^^,^^b^	<0.0001
1–5	18160	42.5	50.2^a^^,^^c^	
>5	6079	14.2	56.3^b^^,^^c^	

*All variables concerning use of other medications than the ACE-inhibitor refer to the year prior to the study period

^a,b,c,d^ Groups assigned with the same letter are significantly (p<0.05) different from each other in pairwise comparisons within each variable.

### Refill adherence

The mean CMA for the entire study population was 53.5% (SD 40.1) (Table 2). CMA was higher among those who were exposed to switching products than among those who were not. CMA also varied by socioeconomic characteristics and previous CVD as well as hospitalisation (Table 2).

Those who used more than five other medications during the year prior to the study period had a lower CMA than those who did not (Table 2). The adjusted CMA was higher among those exposed to switching of the ACE-inhibitor (Table 3). There was no statistically significant difference in the adjusted CMA depending on the number of other medications they had used, neither among those who had switched products nor among those who hadn’t switched products.

**Table 3:** Refill adherence (expressed as mean CMA and 95% confidence intervals) in relation to exposure to switching of the ACE-inhibitor and use of multiple other medications (n = 42735 in crude values and n = 25623 for adjusted values).

Exposure	Switched products	Did not switch products
**Crude values:**		
0–5 other medications	CMA = 80.7%^a^^,^^b^	CMA = 22.8%^a^^,^^c^^,^^d^
	(95% CI 80.3–81.2)	(95% CI 22.3–23.3)
	n = 14606	n = 11217
>5 other medications	CMA = 81.6%^c^^,^^e^	CMA = 26.7%^b^^,^^d^^,^^e^
	(95% CI 81.0–82.2)	(95% CI 26.1–27.3)
	n = 7266	n = 9646
Adjusted values*:		
0–5 other medications	CMA = 75.3%^a^^,^^b^	CMA = 14.7% ^a^^,^^c^^,^^d^
	(95% CI 73.6–77.1)	(95% CI 13.0–16.5)
>5 other medications	CMA = 73.3%^c^^,^^e^	CMA = 15.5%^b^^,^^d^^,^^e^
	(95% CI 71.4–75.3)	(95% CI 13.7–17.5)

ANOVA and ANCOVA with post hoc test Tukey was used to detect differences between groups.

^a,b,c,d,e^ Groups assigned with the same letter are significantly (p<0.05) different from each other when pairwise comparisons were conducted.

* Adjusted for sex, age, educational level, household income, country of birth, use of other CVD medication, use of diabetes medication, number of substitutions on other medications, hospitalisation and previous cardiovascular diagnosis

A multiple linear regression showed that switching products was positively associated with CMA whereas the number of other medications was not associated with CMA (Table 4). Statistically significant covariates were age, hospitalisation and a previous diagnosis of CVD. There was no colinearity between the included variables. The β-value of switching products was similar in the univariable model (β = 56.4, 95% CI 55.9–57.0) and in the multiple model (β = 59.4, 95% CI 58.7–60.0).

**Table 4:** Multiple Linear regression with Continuous measure of Medication Acquisition (CMA) in percent as dependent variable (n = 25623).

Variable	B	95% CI (LCL;UCL)	p-value
**Switching products (yes)**	59.4	58.7; 60.0	<0.001
**Number of other medications used (>5)**	-0.5	-1.2; 0.3	0.252
**Sex**			
Men	Ref.		
Women	-0.7	-1.4;-0.1	0.035
**Age**			
< 45	Ref.		
45–54	2.4	0.9;3.9	0.002
55–64	3.7	2.3;5.1	<0.001
65–74	4.5	3.1; 5.9	<0.001
≥75	6.1	4.3;7.8	<0.001
**Educational level**			
1^st^ quartile (lowest)	Ref.		
2^nd^ quartile	0.4	-0.4;1.1	0.342
3^rd^ quartile	1.5	-0.5;3.5	0.148
4^th^ quartile	0.6	-0.4;1.6	0.237
**Household income**			
1^st^ quartile (lowest)	Ref.		
2^nd^ quartile	0.4	-0.6;1.5	0.392
3^rd^ quartile	1.2	0.2;2.2	0.019
4^th^ quartile	2.9	1.8;3.9	<0.001
**Country of birth**			
Sweden	Ref.		
Nordic countries	-0.7	-2.1;0.7	0.304
Europe	-3.5	-4.9;-2.1	<0.001
Rest of the world	-3.5	-5.2; -1.8	<0.001
**Use of other cardiovascular medication**	-0.0	-0.8; 0.8	0.932
**Use of diabetes medication**			
None	Ref.		
Oral antidiabetics only	2.5	1.3;3.7	<0.001
Oral antidiabetics and Insulin	3.1	1.3; 4.9	0.001
Insulin only	0.9	-0.6;2.5	0.227
**Number of substitutions on other medications**			
0	Ref.		
1–5	0.3	-0.5;1.1	0.405
>5	2.7	1.5; 3.8	<0.001
**Hospitalisation**	3.7	2.8;4.6	<0.001
**Previous cardiovascular diagnosis**	3.3	2.2;4.4	<0.001

All variables were checked for colinearity and had a maximum VIF of 4.34

### Sensitivity analysis

When only individuals with at least two filled ACE-inhibitor prescriptions during the study period were included (n = 24510), CMA increased to 78.0%. The direction of the results did, however, not change. CMA was higher in the groups exposed switching products, compared to those not exposed to switching products but did not differ by use of multiple medications (Table 5).

**Table 5:** Sensitivity analyses of refill adherence, (mean CMA), adjusted for potential confounders*, when restricting the study population to those who a) purchased ACE-inhibitors at least twice (n = 13360), and b) those who did not switch between ACE-inhibitors with different active ingredients during the study period (n = 25175), respectively.

	Switching products	No switching of products
At least two ACE-inhibitor purchases*		
0–5 other medications	CMA = 71.5%^a^^,^^b^	CMA = 45.3%^a^^,^^c^^,^^d^
>5 other medications	CMA = 69.8%^c^^,^^e^	CMA = 47.4%^b^^,^^d^^,^^e^
**No switch between ACE-inhibitors**		
0–5 other medications	CMA = 75.8%^a^^,^^b^^,^^c^	CMA = 14.8%^a^^,^^d^^,^^e^
>5 other medications	CMA = 74.0%^b^^,^^d^^,^^f^	CMA = 15.6%^c^^,^^e^^,^^f^

ANCOVA with post hoc test Tukey was used to detect differences between groups.

*Adjusted for sex, age, educational level, household income, country of birth, use of other CVD medication, use of diabetes medication, number of substitutions on other medications, hospitalisation and previous cardiovascular diagnosis.

^a,b,c,d,e,f^ Groups assigned with the same letter are significantly (p<0.05) different from each other in pairwise comparison.

When the study population was limited to individuals who had not used several different substances of ACE-inhibitors during the study period (n = 41942), the CMA for the remaining study population was unchanged (53.2%). As with the previous sensitivity analysis, this alteration of the study population did not change the direction of the results (Table 5).

When we altered the definition of switching ACE-inhibitor, all participants were considered exposed to switching. It was thus not possible to conduct any analyses comparing the exposed and the unexposed.

As shown in Table 6, there was a downward trend in mean CMA with increasing time from the index date to the time of the first switching of products. This was consistent in both individuals with and without use of multiple medicines before index date. When time to first switching of products was included in the multiple linear regression model, the regression coefficients for switching products increased (Table 7) as compared in the model without this variable (Table 4). There were only minor changes in the regression coefficients for the other variables.

**Table 6:** Sensitivity analyses of refill adherence (mean CMA± standard deviation, SD) in relation to time from index date to the first occasion of switching products by number of other medications among those experiencing switching products (n = 21870).

	≤4 months	4–8 months	8–12 months	>12 months
**0–5 other medications**	82.9±27.3%	85.7±22.2%	74.9±26.2%	59.1±29.0%
	n = 9253	n = 2873	n = 1190	n = 1290
**>5 other medications**	86.7±24.5%	86.2±22.2%	73.6±27.0%	59.2 ±29.2%
	n = 3608	n = 1919	n = 827	n = 912
**Total**	84.0±26.5%	85.9±22.2%	74.4±26.6%	59.1±29.1%
	n = 12861	n = 4792	n = 2017	n = 2202

**Table 7:** Multiple Linear regression with Continuous measure of Medication Acquisition (CMA) in percent as dependent variable including time to first switch of ACE-inhibitor product (n = 34711).

Variable	B	95% CI (LCL; UCL)	p-value
**Switching products**	69.4	68.5;70.2	<0.001
**Number of other medications used**	-0.3	-1.1;0.4	0.394
**Time to first generic substitution (days)**	-0.1	-0.1; -0.1	<0.001
**Sex**			
Men	Ref		
Women	-0.9	-1.6;-0.2	0.007
**Age**			
< 45	Ref		
45–54	2.3	0.8;3.7	0.002
55–64	3.6	2.2;5.0	<0.001
65–74	4.3	3.0;5.7	<0.001
≥75	6.1	4.3;7.8	<0.001
**Educational level**			
Elementary school	Ref		
High school	0.3	-0.5;1.0	0.477
Higher education≤2 years	1.3	-0.6;3.3	0.182
Higher education>2 years	0.5	-0.4;1.5	0.288
**Household income**			
1^st^ quartile (lowest)	Ref		
2^nd^ quartile	0.3	-0.7; 1.3	0.502
3^rd^ quartile	0.9	-0.1; 1.9	0.072
4^th^ quartile	2.3	1.3; 3.4	<0.001
**Country of birth**			
Sweden	Ref		
Nordic countries	-0.5	-1.9;0.8	0.444
Europe	-2.2	-3.6;-0.9	0.001
Rest of the world	-2.5	-4.2;-0.8	0.004
**Use of other cardiovascular medication**	-0.1	-0.8;0.7	0.876
**Use of diabetes medication**			
None	Ref		
Oral antidiabetics only	3.0	1.8; 4.2	<0.001
Oral antidiabetics and Insulin	3.3	1.6;5.1	<0.001
Insulin only	1.8	0.3;3.3	0.020
**Number of substitutions on other medications**			
0	Ref		
1–5	0.2	-0.7;0.9	0.749
>5	2.1	-1.0;3.2	<0.001
**Hospitalisation**	3.3	2.4;4.1	<0.001
**Previous cardiovascular diagnosis**	3.3	2.3;4.4	<0.001

## Discussion

### Discussion of results

Around half of the participants were exposed to switching ACE-inhibitor product, mainly due to generic substitution, during the study period. Refill adherence was higher among those exposed to switching products than among those not. Among those exposed to switching ACE-inhibitor, there was no difference in refill adherence if they had also used more than five different other medications. Refill adherence was also higher with higher age, previous hospitalisation and previous cardiovascular diagnosis. It was highest in the group with the lowest household income and lowest among those born outside the Nordic countries.

Contrary to what could be expected from previous studies, based on interviews with patients or pharmacists [10–13], refill adherence was higher among those who were exposed to switching within the same active substance, than among those who were not. Our findings are in line with some previous register based studies [15–17], although others have reported conflicting results on this matter [9]. One possible reason explaining the result found in this study is that patients who experience substitution may receive more attention and support from the pharmacy staff when purchasing their medication than patients not experiencing switching products. In addition, a reason for switching brands could be that the patient experienced adverse reactions from the first product and this is resolved when switching to another product, which may affect refill adherence in a positive direction. Another possible mechanism could be that the generic substitution does not affect the tendency to purchase medication from the pharmacy, but it could cause confusion when the patient comes home and tries to use the medication according to the prescribers recommendations. Such difficulties cannot be captured by refill adherence.

Use of multiple medications was regarded as a factor that could complicate the medication use for the patient and thus lead to lower adherence. Previous studies reported both negative associations between the number of medications used and adherence [3, 29] and positive association between number of medications and adherence [30–32]. We did not detect any significant differences in refill adherence by use of multiple medications. A possible explanation for this may be that patients using a large number of medications have developed routines for collecting their medications at the pharmacy, thus contributing to a similar refill adherence. It is well known that time is an important factor for individuals to adopt a health behaviour [33]. It is also possible that patients prescribed multiple medications feel more personally vulnerable to the consequences of disease and therefore may be more adherent to prescribed medicines compared with patients with fewer prescriptions [30].

The results also indicate higher refill adherence among those with previous CVD. Patents with hypertension experience none or few symptoms and because of this they may not realize the importance of the medications. The potential risk may, however be more evident for patients who have already experienced a cardiovascular event or been hospitalised. Refill adherence also varied with sociodemographic factors. The most pronounced factor was age, with the highest adherence among the eldest and the lowest among the youngest. Previous studies reported similar age differences [31, 34, 35]. There was also a difference in refill adherence by household income. Those with a low income had the highest adherence. The construction of the Swedish pharmaceutical benefits scheme partly explains this, as the patient pays a moderate sum out of pocket. Affordability may thus not be the foremost explanation behind socioeconomic differences. Rather it could be a matter of health literacy or autonomy in relation to the prescriber. Another potential explanation is that the lowest income group includes a large part of the retirees and adherence increases with age. Those born in Sweden had a higher refill adherence than those born outside the Nordic countries. These differences are important aspects of inequality of health care. As individuals with foreign background are almost always underrepresented in surveys, register based studies are an important tool to capture such differences. However, it should be kept in mind that the registers only contain data on individuals who are registered as residents in Sweden, thus excluding those residing temporarily in Sweden.

For some patients, CMA exceeds 100% indicating that they has purchased more medication than needed, according to the dosage label, during the study period. The Swedish pharmaceutical benefits scheme allows patients to make the next purchase when two thirds of the amount previously purchased is consumed, thus enabling stock piling. A CMA exceeding 100% may also indicate that some patients are overusing medication. Interview studies have suggested that patients may accidentally use several products with the same active ingredient at the same time after generic substitution [11]. As this would not imply a higher degree of adherence we chose to limit the maximum value of CMA to 100%.

### Methodological discussion

One of the main strengths of the study is the large sample size, including everyone who purchased an ACE-inhibitor in Sweden during the study period. Due to this, parametric methods were considered to be suitable in the statistical analyses. The residual plots also showed that a linear model was a suitable analysis. Individuals receiving multi-dose dispensed drugs during the study period were excluded, as medications are generally automatically dispensed to them and would provide an artificially regular adherence pattern. All data included in the analyses were on an individual level. There is no information on medicine use during hospitalisation in the SPDR. By linking the SPDR to the National Patient Register we could avoid misclassification of time with no refilled prescription due to hospitalisation. Missing values occurred, as in all routinely recorded data, although the impact of this was considered to be manageable. It is however possible that bias may be present and certain patient groups may be either over- or underrepresented due to missing data on covariates, in particular the socioeconomic characteristics. Educational level is largely missing for elderly patients and patients born abroad who immigrated to Sweden decades ago when this was seldom registered and who has since not been studying in Sweden.

Another strength is that the treatment periods for each purchase was based on the prescribed daily dose, instead of technical measures as defined daily doses. An algorithm was developed to interpret the dosage instructions [23], which are included in the SPDR as free text. This allowed us to consider variation of dosages over time, e.g. titrations. Refill adherence was measured using CMA treated as a continuous variable, making full use of the data and avoiding arbitrary cut offs. In a previous publication we have concluded that CMA is a suitable measure for assessing refill-adherence in a Swedish context [23]. It is particularly useful when long-term continuous medication treatment is investigated [36]. In order to isolate the effect of switching products on refill adherence, only new users of ACE-inhibitors were included, thus obtaining a more homogenous population and avoiding previously developed patterns of adherence. The length of the observation period during which CMA is assessed is also important and may affect the estimates of CMA. A short period will contribute to higher CMAs whereas CMA tends to stabilize once time exceeds 18 months [23].

Our definition of switching products was chosen since the aim includes generic substitution and switching products from a patient perspective. The most cumbersome aspect of generic substitution for each patient is most likely the switching between different products at every purchase. In Sweden, the patient is dispensed the cheapest substitutable alternative to the prescribed drug that is available at the pharmacy at each purchase. Because of this, a patient using chronic medication, such as ACE-inhibitors, could end up receiving a different product at each purchase. For the individual patient, the switching between different products is probably more notable than the difference between using brand or generic products. Since the main outcome of this analysis is the patients’ behaviour (adherence to medication treatment) we found it appropriate to focus on aspects that are most notable to the patient. As we focused on the situation where the patient receives a different product than the one he or she received at the previous fill, we chose not to use the variable in SPDR denoting if a product was substituted compared to what was written on the prescription. The study population included new users only and since most prescriptions in Sweden are electronically transferred to the pharmacy the patient never sees the prescription before coming to the pharmacy. Thus we considered that receiving different products at different purchases would be more important from the patient perspective. To assess to what extent this definition affected the results we conducted a sensitivity analysis using the variable on generic substitution in the SPDR to define exposure. However for the medicines purchased prior to index date we did not know whether the patient was a new user or not and therefore choose to use the variable in SPDR. We also conducted a sensitivity analysis to assess if the difference in CMA between those with and without switching of products was affected by the time elapsed from the index date to the first occurrence of switching products. The results of this analysis show that those who were exposed to switching products from the beginning of the study period had a higher CMA than those who had their first occurrence of switching products later on. This suggests that the finding in the main analysis, i.e. a higher CMA among those exposed to switching products at any time, may be underestimated.

The study is entirely based on data from Sweden and because of this, the results may have limited generalizability in other countries with very different systems for generic substitution and coverage for medications.

## Conclusions

The results of this population-based study indicate a positive association between refill adherence and switching products among new users of ACE-inhibitors in Sweden. The refill adherence was higher among those exposed to switching products compared to those not exposed, regardless of whether they had used multiple other medications or not.

The influence of other factors such as previous cardiovascular diagnosis and previous hospitalisation suggest that the more experience from medication use and health care the patient has, the more adherent he or she is. However, more research is needed on the reasons behind these associations and whether these remain when using other measures and data to study adherence.
